# Quantitative assessment of regional variation in tissue clearing efficiency using optical coherence tomography (OCT) and magnetic resonance imaging (MRI): A feasibility study

**DOI:** 10.1038/s41598-019-39634-z

**Published:** 2019-02-27

**Authors:** Kwangyeol Baek, Sunwoo Jung, Junwon Lee, Eunjung Min, Woonggyu Jung, Hyungjoon Cho

**Affiliations:** 1 Department of Biomedical Engineering, Ulsan National Institute of Science and Technology (UNIST), Ulsan, South Korea; 2 The Rowland Institute at Harvard, 100 Edwin H. Land Blvd, Cambridge, MA 02142 USA

## Abstract

Tissue clearing has gained attention as a pioneering research tool for imaging of large tissue samples. This technique improves light transmission by reducing light scattering within tissues, either by removing lipids or by replacing water with a high refractive index solution. Although various clearing techniques have been developed, quantitative assessments on clearing efficacy depending on tissue properties are rare. In this study, we developed the quantitative mapping of regional clearing efficacy using mean free path in optical coherence tomography (OCT) and proton density in magnetic resonance imaging (MRI), and demonstrated its feasibility in the brain sample with four representative clearing techniques (benzyl alcohol and benzyl benzoate [BABB], Clear^T^, Scale, and passive CLARITY technique [PACT]). BABB (solvent-based clearing), involving both refractive index matching and lipid removal, exhibited best optical clearing performance with the highest proton density reduction both in gray and white matter. Lipid-removing techniques such as Scale (aqueous hyperhydration) and PACT (hydrogel embedding) showed higher clearing efficiency in white matter than gray matter in accordance with larger proton density increase in white matter. For Clear^T^ (aqueous-based simple immersion), we observed lowest clearing efficiency in the white matter as well as poor lipid removal reflected in low proton density reduction. Our results showed the feasibility of the regional mapping of clearing efficacy and correlating optical transparency and proton density changes using OCT and MRI from existing tissue clearing techniques. This novel quantitative mapping of clearing efficacy depending on tissue types and clearing methods may be helpful in the development of optimized clearing methods for different biological samples.

## Introduction

Light microscopy is one of the most prominent tool in neuroscience as it allows the study of the structure and function of the brain. During the last few decades, optical microscopy has integrated state of the art imaging modalities in terms of resolution, contrast, sensitivity, and image-acquisition time. However, the attainable imaging depth is still limited to a few hundreds of micrometers, due to multiple light scattering in the turbid brain tissue. Hence, optical imaging studies of the brain often involve imaging of multiple sections of the sample and subsequent volumetric reconstruction, which is a labor-intensive and time-consuming process. The techniques for optical clearing of biological tissues have helped in minimizing light scattering during imaging, thus enabling researchers to overcome the aforementioned limitations and study large-volume biological tissues^1,2^. To date, various tissue clearing techniques have been developed^3^. Tissue clearing techniques usually reduce inhomogeneity in optical properties in the tissue by (1) removing lipids, a main source of light scattering in the turbid tissue, and/or (2) replacing water in the sample with the clearing solution having a high refractive index. For example, “solvent-based clearing” such as BABB^4^ removes out water and lipids from the sample and homogenize the refractive index using the clearing solution. In contrast, “simple immersion” such as Clear^T^ ^5^ does not remove out lipids but replacing water in the sample with a clearing solution that matching average refractive index of the biological tissues. “Aqueous hyperhydration” techniques such as Scale^6^ utilize detergent-based removal of lipid in conjunction with urea-mediated hydration. “Hydrogel embedding” techniques such as PACT^7^ enable more severe detergent treatment to remove lipids and then immerse the cleared sample in a refractive index matching solution.

Performance of these clearing techniques might vary depending on the constitution of the biological tissue. For example, tissue clearing efficiency can vary across gray matter and white matter regions of the brain, since white matter region contains the lipid-rich myelin sheaths. Although it is often stated that the light scattering at the interface of refractive index mismatch determines the turbidity of the tissue, a uniform density of scatterers achieved by lipid-removal is also a key factor in minimizing the lateral scattering of light^3^. Thus, tissue clearing methods involving lipid removal can be much more efficient in dealing with heterogeneous distribution of lipids within white matter region. This type of regional variation in clearing efficacy depending on tissue properties might be critical to optimize clearing methods in individual application. However, there are few imaging methods to quantitatively assess the efficacy of clearing methods across regions in a biological sample.

One of candidate imaging modalities is optical coherence tomography (OCT), a high-resolution tomographic imaging technique which relies on the intrinsic scattering properties of biological tissues to generate imaging contrast^8^. In addition, it has been verified that the optical properties of a biological tissue^9,10^, especially the attenuation coefficient^11–13^, can be quantified by analyzing OCT signals. In previous studies, the use of the attenuation coefficient, as determined by OCT, allowed the discrimination of atherosclerotic plaques in the human carotid artery^14,15^, of tumorous tissue in the human brain^16^, of apoptosis/necrosis in human fibroblasts^17^, and of the laminar structure in the rodent cortex^18^. Recently, several studies have reported methods of evaluating and quantifying the efficacy of the existing clearing techniques. The most common approach is the measurement of light transmittance in the tissue before and after clearing^19,20^. In one study, the attenuation coefficients were quantified and the efficiencies of three different clearing techniques, including BABB and passive CLARITY (PACT), were compared^21^. These studies demonstrated that OCT is suitable for the quantitative monitoring of optical attenuation in biological tissues during clearing. However, these studies did not analyze the quantitative and regional variations in light attenuation and those in the density of the scatterers within the brain.

In addition, magnetic resonance imaging (MRI) can provide important information about the regional difference in tissue responses during clearing processes, such as hyperhydration associated with lipid removal. For example, a recent study demonstrated the large reduction in tissue-dependent MRI contrasts in the brain after CLARITY (PACT), and identified lipids as the source of MRI contrast in structural brain imaging^22^. However, to the best of our knowledge, the regional relationship between MRI parameters and optical clearing efficacy has not yet been quantitatively established. Proton density is a quantitative MRI assessment of hydrogen atoms (waters) in the sample, and can be used to estimate hyperhydration or dehydration associated with tissue clearing techniques. Subsequently, the quantitative comparison of proton densities with respect to optical transparency would facilitate the evaluation and understanding of clearing efficacies of the various clearing methods in relation to tissue characteristics.

In this study, we demonstrate the quantitative mapping of clearing efficacy in the brain sample using OCT and MRI. In OCT, we proposed the mean free path (MFP), the average distance that a photon travels between successive scattering events, as a measure of optical transparency, and mapped the regional variation in optical clearing efficiency in the mouse brain slices. Using MRI relaxometry, we mapped the regional proton densities before and after each clearing process, and monitored the regional relationship between the increased optical transparency (OCT-based MFP) and the change in the water content (MRI-based proton density). We examined the feasibility of our methods with four conventional clearing techniques: BABB, Clear^T^, Scale, and PACT. These are chosen as representative techniques from four large categories of clearing methods, which are “solvent-based clearing”, “aqueous simple immersion”, “hyperhydration”, and “hydrogel embedding”. We hypothesized that lipid-rich white matter region might have poor optical transparency than gray matter at baseline and lipid-removing clearing methods (Scale and PACT) will be more efficient in white matter region. We expected that Clear^T^ might have limited efficacy in white matter region with excessive lipid distribution. Finally, we hypothesized that proton density measurement in MRI might reflect optical clearing efficacy measured in OCT in a quantitative manner.

## Methods

### Tissue Preparation and Clearing

All animal procedures were approved by the *In Vivo* Research Center at Ulsan National Institute of Science and Technology (UNIST) and carried out in accordance with the Institutional Animal Care and Use Committee standards (UNISTIACUC-15-27). We obtained the brains from eight-week-old male C57BL/6 mice. After being deeply anesthetized by intraperitoneal injection of tiletamine/zolazepam (Zoletil; 30 mg/kg) and xylazine (Rompun; 10 mg/kg), the mice were transcardially perfused with 30 mL of phosphate buffer saline (PBS), followed by 60 mL of 10% neutral buffered formalin (NBF). The brains were dissected out and incubated in 10% NBF for 1 day at 4 °C. Using a vibratome (VT 1000S, Leica), the brains were cut into 3-mm-thick coronal slices with highly uniform surfaces. The brain slices were then cleared, each with one of the four different clearing protocols. For the Clear^T^ experiment, we immersed the brain slices in a series of solutions of increasing formamide concentrations (20%, 40%, 60%, 80%, and 100%) for 12 hours each, except for the last step which took overnight^5^. For BABB clearing^4^, the brain slices were first dehydrated in ethanol (100%), and were then transferred into a solution containing a 1:2 mixture of benzyl alcohol and benzyl benzoate. For Scale clearing^6^, the brain slices were submerged in a solution of 4 M urea, 10% glycerol, and 0.1% Triton X-100, for 1 month. We stored the cleared tissues in each clearing solution, before and during optical image acquisition. For PACT^7^, we hybridized the brain slices with hydrogel and then immersed them in the PACT solution for 4 weeks. Hydrogel monomer is composed of 4% acrylamide, 0.25% initiator, and 4% paraformaldehyde, in PBS. For hydrogel monomer infusion, the brain slices were immersed in the monomer solution for 3 days at 4 °C. We then increased the temperature while immersing the infused brain slices in the monomer solution for polymerization. After tissue hybridization with the hydrogel monomer, the brain slices were cleared with the PACT solution (8% sodium dodecyl sulfate) at 20 °C for 4 weeks. In the present study, we focused on lipid removal effect of PACT^7^, thus omitted the final step of incubating the PACT-cleared sample in a refractive index matching solution such as Focus Clear. It was intended to directly compare the proton density measurement in MRI and the PACT clearing efficacy in OCT. We need to embed the PACT cleared brain sample in 1.5% agarose gel in our MRI experiment (see D. Magnetic Resonance Imaging Analysis) due to the fragileness of the cleared sample, which prevented use of a refractive index matching solution in PACT clearing.

### Spectral-Domain Optical Coherence Tomography and Wide-Field Imaging

In Supplementary Fig. 1A, a schematic illustration of the spectral-domain OCT (SD-OCT) system is presented. The system uses a super-luminescent diode (EXS210046-02, Exalos) which operates at a center wavelength of 1,310 nm with a bandwidth of 70 nm, providing an axial resolution of 10.7 µm in air. A 2 × 2 fused fiber optic coupler divides the incident light towards the sample and the reference paths. In each path, objective lenses with a focal length of 40 mm and numerical aperture of 0.085 are used, resulting in a lateral resolution of 5.7 µm. In the sample path, a set of dual-axis galvanometer scanners (GVS012, Thorlabs) is used to laterally scan the incident light over the specimen. The lights reflected from each path are then combined at the fiber coupler and interference occurs. A spectrometer consists of a transmission grating (Wasatch Photonics), a lens, and a line scan camera (SU1024-LDH2, Sensors Unlimited) with a resolution of 1024 pixels. The interference is acquired by the camera, and processed with software written in LabVIEW, supporting standard SD-OCT signal processing such as wave number linearization, dispersion compensation, and inverse Fourier transformation. The imaging acquisition time for volumetric scanning of 500 × 500 × 512 pixels corresponding to 2.5 × 2.5 × 1.7 mm^3^ was 12.5 s. The system had a sensitivity of 98.6 dB. We designed a customized sample holder that was mounted on the set of two linear motorized stages to flatten the top surface of the brain slices. The samples were immersed into the PBS buffer solution (OCT before clearing) or final clearing solutions (OCT after clearing). We also utilized a fully automated wide-field imaging technique that enables mosaic imaging of sliced tissue^23^. The overall imaging area was 12.5 × 10 mm^2^ (horizontal × vertical). The full field of view is made up of 20 mosaic images without overlap between adjacent images.

### Analysis of Optical Properties and Tissue Deformation

To investigate the induced size alterations due to the effect of clearing, we prepared a brain slice and cut it along the mid-sagittal plane. One half of the slice was immersed in PBS, and the other half was subjected to clearing. After the clearing procedure had finished, the slices were imaged by OCT before histological processing. Following this, the slices were dehydrated, embedded in paraffin, and sectioned into 4-μm-thick coronal slices using a microtome (RM2255, Leica). For histological staining, hematoxylin and eosin were used. The stained slices were imaged using a microscope equipped with a 20× objective lens.

In order to obtain quantitative measurements regarding physical alterations due to the clearing effect, we acquired 3D OCT images and analyzed them on the en-face view after intensive image processing. In particular, the OCT images were initially filtered with a 3 × 3 median filter to reduce shot noise, and the resulting images were converted into binary values using Otsu’s global thresholding method. The Sobel operator was then applied on the binary image to define its boundary. Using the boundary information, we measured the clearing-induced shift of the boundaries. The deformation analysis by means of boundary comparison was performed in three steps. First, each extracted boundary, before and after clearing, was aligned based on their centroid and principal axes. Next, we determined the intersecting points between the boundaries and the straight lines, spaced 1 degree apart. We assumed that the cleared tissue has the tendency to deform towards a certain direction. Last, we measured the distance between two intersecting points on the same straight line, and defined this spacing as the shift of the boundaries.

To evaluate the attenuation coefficient, we calculated the natural logarithm of the OCT signal by the following equation:

1 $$\mathrm{ln}\,I\,({\rm{z}})=\,\mathrm{ln}\,{\rm{\rho }}-\mu \,{\rm{z}}$$

where I(z) is the light intensity at distance z, ρ is the reflectivity on the tissue surface and μ is the attenuation coefficient. To measure the optical properties described below, the OCT signals in the respective regions of interest (ROIs) were averaged and then analyzed. The reflectivity is defined as the OCT signal obtained at the top surface of the tissue. Then, we performed the linear squares fit on the part of the logarithmic signal where the signal was attenuated to 37% (1/e) of that on the surface. The attenuation coefficient corresponds to the slope of the linear squares fit^18^. Lastly, we defined the MFP as the inverse of the attenuation coefficient.

### Magnetic Resonance Imaging Analysis

We conducted MRI experiments on whole-brain samples to assess the changes in the chemical constitution of the brain samples after clearing. Prior to tissue clearing, the whole-brain samples were embedded in 1.5% agarose gel for MRI scan, and afterwards they were removed from the gel for tissue clearing. After the required period of tissue clearing (4 weeks for Scale and PACT, 2 weeks for Clear^T^, and 1 week of dehydration for BABB), the brain samples were prepared for the post-clearing MRI scan. The brain samples that were cleared with water-based solution (Scale [n = 3] and PACT [n = 3]) were embedded in 1.5% agarose gel which worked as a structural scaffolding as well as a reference for baseline measurement of MRI signal intensity (see below). The samples cleared with Clear^T^ (n = 4) or BABB (n = 3) were immobilized in a syringe filled with the clearing solution, and the syringe was placed in the 1.5% agarose gel for MRI scan. The 1.5% agarose gel was made in same condition across the samples before and after clearing so that it can be used as a baseline measurement for normalizing MRI signal intensity across samples. The agarose gel was also wrapped with plastic film to avoid drying during the MRI experiment.

All MRI experiments were performed in the Bruker 7 T small-bore scanner operated by a Bruker Paravision 6.0 console. Three whole brain samples, for each clearing method, were scanned pre- and post- clearing. We used the multi-slice multi-echo sequence, along echo time (TE) of 10, 20, 30, …, 100 ms. This multi-echo acquisition is ideal for fitting the T_2_ decay curve along multiple TEs, as is shown in following equation:

2 $${\rm{S}}({\rm{n}}{\rm{E}},{\rm{T}}{\rm{E}})={{\rm{S}}}_{0}{{\rm{e}}}^{(-{\rm{n}}{\rm{E}}\times {\rm{T}}{\rm{E}}/{\rm{T}}2)}$$

where nE and TE are the number of echoes and echo time, respectively. We used the following imaging parameters: repetition time (TR) = 3000 ms, TE = 10 ms, nE = 10, matrix size = 256 × 256, field of view = 15 × 15 mm^2^, slice thickness = 0.4 mm, slice gap = 0.1 mm, number of slices = 25 (interleaved acquisition), voxel resolution = 58 × 58 × 500 μm^3^, number of averagings = 30, scan time = 6 h 24 min.

Using MATLAB (Mathworks, Natick, MA), we calculated the proton density signal (S0) from the MRI signal intensity in each voxel. S0 was set as the MRI signal amplitude at TE = 0 ms which is extrapolated from the T_2_ decay curve and is proportional to the hydrogen content in each individual voxel. We normalized the S0 in the brain samples to the corresponding S0 in the 1.5% agarose gel area (manually drawn ROIs) for comparison across multiple samples. In the present study, the normalized S0 in the brain samples was used as the measurement of proton density (i.e. hydrogen index), a quantitative parameter of the hydration or dehydration effect in tissue clearing methods.

We visualized the changes in the hydrogen index (normalized S0) in the brain before and after each method of tissue clearing. In addition, we manually drew ROIs in the cerebral cortex and in the corpus callosum in each sample in order to compare the changes in MRI parameters in the gray and white matter, respectively. We calculated the average S0 values for the cerebral cortex (gray matter) and the corpus callosum (white matter) in each slice, and compared the changes in these values after tissue clearing using a two-sample t-test.

## Results

### Observation of Tissue Deformation Upon Clearing

**Figure 1:**
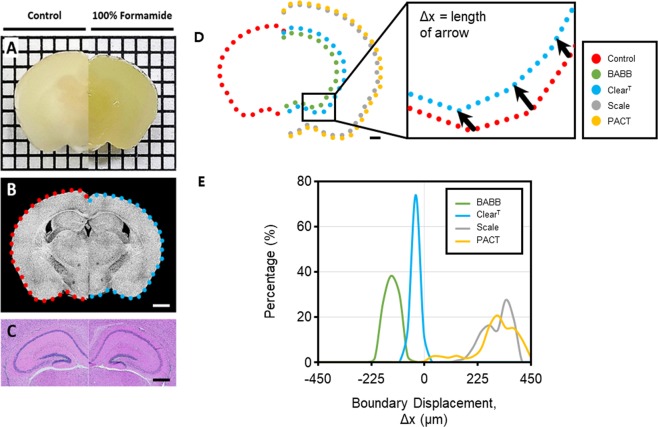
Images of a coronal brain slice (left, control; right, formamide treated) taken by (**A**) digital camera, (**B**) optical coherence tomography (OCT), and (**C**) wide field microscope (hematoxylin and eosin histological staining). In (**A**), the tick marks represent 2 mm. In (**B**,**C**), scale bars represent 2 mm and 1 mm, respectively. (**D**) Illustration of the overlapped boundaries of brain slices after three different clearing techniques: benzyl alcohol and benzyl benzoate (BABB), Clear^T^ and Scale. Scale bar represents 2 mm. (**E**) Histogram of the relative tissue volume changes after each of the four clearing techniques.

Figures 1A and B show microscopic photos and top view of 3D OCT images of a brain slice before and after Clear^T^, respectively. As presented in both figures, the opaque brain tissue became semi-transparent after Clear^T^, while the size of the tissue was not retained. The histological staining with hematoxylin and eosin demonstrated that the cellular morphology was well preserved in the sections after tissue clearing (Fig. 1C). Figure 1D shows the overlapped boundaries of brain slices before and after the four different clearing techniques: BABB, Clear^T^, Scale, and PACT, illustrating how their original structures were preserved or deformed through tissue clearing. Among the clearing techniques that are based on refractive index matching, BABB clearing caused tissue shrinkage due to the dehydration step in comparison to Clear^T^ that caused less shrinkage. On the other hand, for the clearing techniques that are based on lipid extraction, both Scale and PACT methods caused tissue expansion due to the hyperhydration that follows lipid removal. The deformation of the tissue was quantitatively analyzed for each clearing technique and illustrated in a histogram (Fig. 1E) showing that Clear^T^ maintained the original shape of the tissue. In contrast, the tissue volume changed in a non-linear fashion on the application of the Scale, PACT, and BABB methods.

### Evaluation of the Effect of Clearing on Oct Signals

Clear^T^ method preserved the original shape of the tissue, as aforementioned. Therefore, we applied this approach in order to study the clearing effect by measuring the changes in the optical properties upon clearing of the brain slices. We investigated the OCT signals along the depth of the tissue, while varying the concentration of Clear^T^ solution. Supplementary Fig. 2A displays 3D OCT images of a ROI drawn on a brain slice upon treatment with increasing concentrations of formamide. The OCT image of the control brain slice – not exposed to formamide – presented a very narrow imaging depth. In the brain slices at the different clearing stages (different formamide concentrations) the imaging depth in the cortical and thalamic regions increased by more than two times, allowing imaging of much deeper structures. However, the imaging depth in fibrous regions, e.g., in the corpus callosum, did not increase significantly. Thus, it was apparent that the effect of tissue clearing varies widely across different regions of a single brain slice.

The plotted OCT signals obtained by averaging signals within the ROIs drawn in the cortex (red rectangle of 50 × 50 μm^2^ in the inset of the 2D OCT image; Supplementary Fig. 2B) demonstrate that the attenuation of the OCT signal becomes smaller as the concentration of the clearing solution increased. Therefore, the OCT signals have the potential to be used for the quantitative evaluation of the clearing effect.

### Visualization of the Optical Properties of the Tissue on Oct Images

**Figure 2:**
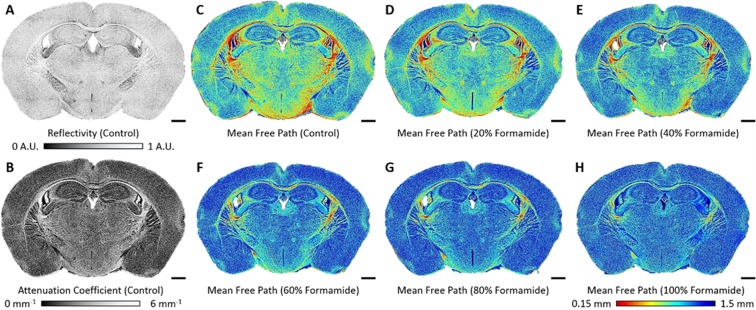
Visualization of the changes in optical properties as tissue clearing renders brain slices transparent: (**A**) reflectivity, (**B**) attenuation coefficient, and (**C–H**) color map of the MFPs. Scale bars represent 1 mm. A.U., arbitrary units.

For the comprehensive analysis of different brain structures, their optical properties were overlaid on the en-face OCT image. Figure 2A,B present the maps of reflectivity and attenuation coefficient of the control brain slice, respectively. The image contrast is more pronounced in the map of the attenuation coefficient than in the reflectivity map, indicating that the attenuation coefficient is suitable for monitoring the clearing effect. To improve contrast visualization we applied a color map for the MFP. Figure 2C–H illustrate the colorized maps of the MFPs on a brain slice, which is being cleared with formamide solutions of increasing concentrations. The cortical and thalamic regions presented high values of MFP (blue color), while fibrous regions, e.g., the corpus callosum, presented low values of MFP (red color). As the maps show, our method can quantitatively and rather accurately visualize the level to which tissue clearing improves imaging depth in relation to the composition of the different brain regions.

### Analysis of the Optical Properties of the Brain Tissue

**Figure 3:**
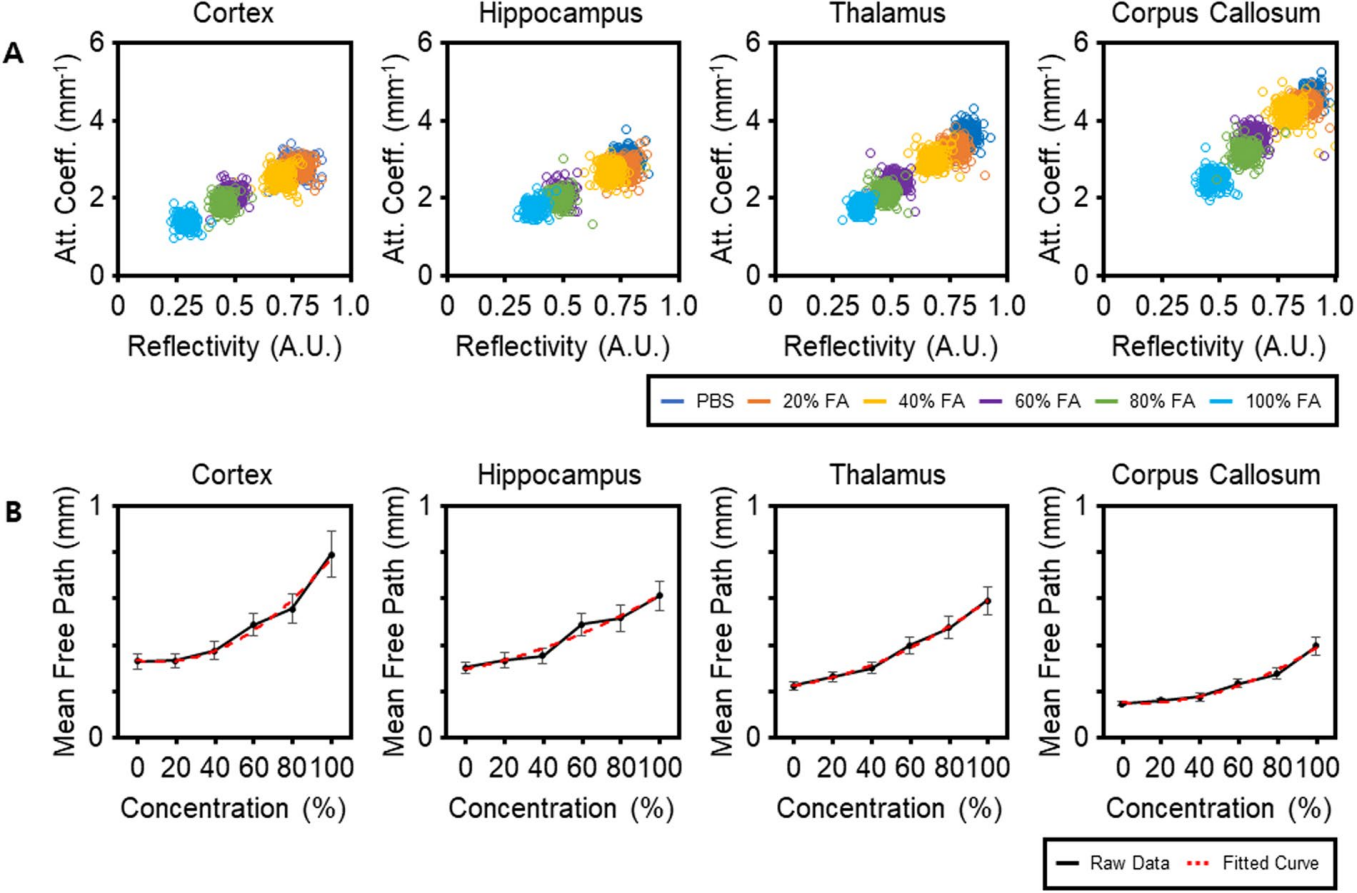
Plots illustrating the changes in optical properties varying with the degree of tissue clearing: (**A**) reflectivity versus attenuation coefficient, and (**B**) the concentration of clearing solutions versus mean free path (MFP). Each circle in (**A**) represents the data obtained from a single position within the regions of interest (ROIs). Red dashed lines in (B) are the second-order fitted curves showing the increasing trend of the MFPs. The MFPs were obtained from the averaged OCT signals of the corresponding rectangular ROIs, indicated by the red rectangle for cortex for example, shown in Supplementary Fig. 2B for each region upon Clear^T^ treatment. Values on plots in (**B**) represent means ± standard deviation (SD). A.U., arbitrary units; FA, formamide; PBS, phosphate buffered saline.

To investigate the changes in the tissue’s optical properties upon clearing, the reflectivity and attenuation coefficient of four different rectangular ROIs (shown in Supplementary Fig. 2B for cortex as red-rectangle) in the cortex, hippocampus, thalamus, and corpus callosum were plotted, as shown in Fig. 3A. For the control (not cleared) brain slice, the attenuation coefficient varied broadly from approximately 3.0 mm^−1^ in the cortex to approximately 5.0 mm^−1^ in the corpus callosum. The reflectivity of the various ROIs had similar values except for that measured in the corpus callosum. During the course of tissue clearing, the decrease in the attenuation coefficient differed significantly in distinct brain regions while the reflectivity was relatively similar. In particular, the attenuation coefficient in the cortex decreased by approximately 50%, whereas it decreased by approximately 46% in the corpus callosum. In contrast, the difference between the decrease in reflectivity in the cortex and in the corpus callosum was insignificant. These results indicate that the attenuation coefficient is a more suitable measure for the evaluation of clearing than is reflectivity. We next analyzed the clearing effect in terms of the MFP in order to provide a more thorough representation. The plots representing the MFP of the ROIs versus the increasing concentration of formamide (Fig. 3B) exhibit a dramatic increase in the MFP. Particularly, as the concentration of the clearing solution rose from 0% to 100%, the MFP in the cortex (the ROI shown in Supplementary Fig. 2B) increased from approximately 0.38 mm to approximately 0.79 mm while the MFP in the ROI in the corpus callosum increased from approximately 0.18 mm to approximately 0.4 mm. Similarly to the results on the attenuation coefficient, these data show that the MFP is strongly influenced by the specific brain region composition and by the degree of tissue clearing. The MFP increase was highest in the cortex, followed by the thalamus, hippocampus, and the corpus callosum. The large increase of MFP might indicate that the tissue contains small amounts of lipids^24^. In other words, Clear^T^ clearing is less effective in lipid-rich regions such as the corpus callosum. These results are in agreement with those reported in other studies^3,5^.

### Proton Density Changes Upon Tissue Clearing

**Figure 4:**
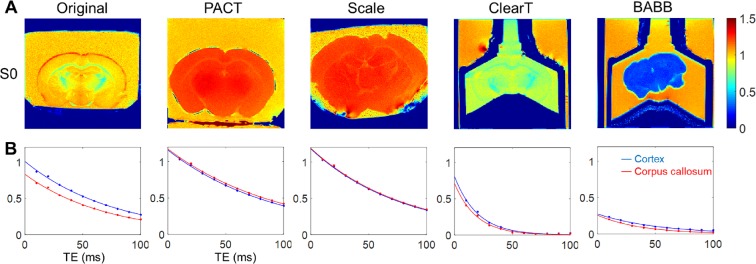
Magnetic resonance imaging (MRI) measurements in the brain samples before and after tissue clearing (Representative samples). (**A**) Hydrogen density index, i.e., proton density signal normalized to the signal of reference 1.5% agarose gel, reflects the relative water content in the samples. (**B**) T_2_ decay characteristics in the cortex and the corpus callosum ROIs. TE, echo time.

To identify water distribution change in distinct types of tissue clearing techniques, we calculated the hydrogen index (i.e., the normalized proton density) in the brain samples, using MRI. As shown in Fig. 4A, PACT and Scale resulted in enlarged brain volume and increase in proton density, i.e., hyperhydration of the brain sample. The proton density after clearing with Scale and PACT exceeded that of the reference 1.5% agarose gel. Dehydration, i.e., reduction in proton density, was apparent upon clearing with Clear^T^ and BABB. After Clear^T^ application, there was a slight shrinkage in the brain volume and a reduction in proton density. BABB clearing resulted in a drastic reduction in both volume and proton density, which is associated with the extensive dehydration. Lower proton density in the corpus callosum (lipid-rich region), as compared to the corresponding measure in the cortex, is apparent in the original brain sample. (Fig. 4A). This regional difference in proton density was preserved in the brain samples cleared with Clear^T^. However, a homogeneous proton density between gray and white matter was observed in the cases of PACT, Scale, and BABB. In summary, regional contrast in proton density (due to lipid contents in white matter) was removed after clearing with PACT, Scale or BABB.

### Comparison of the OCT and MRI Measurements in Four Clearing Techniques: BABB, Clear^T^, PACT and Scale

**Figure 5:**
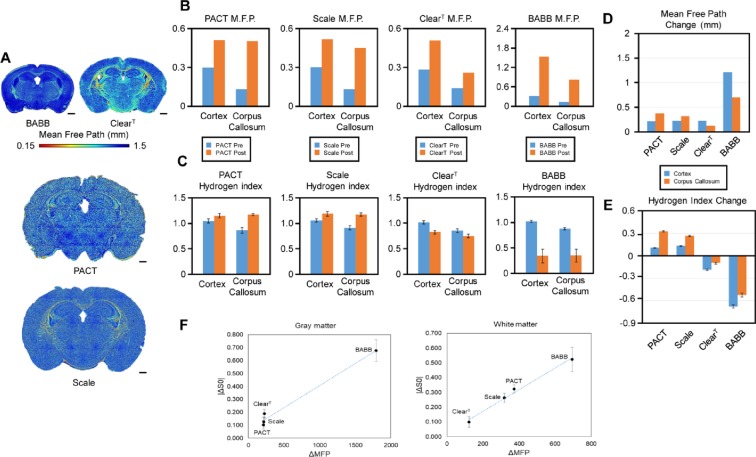
Changes in the optical and chemical properties of the brain samples after tissue clearing. (**A**) Tissue transparency (MFP) across brain regions upon the four different clearing protocols. Scale bars represent 1 mm. (**B**) MFPs before and after application of each of the four tissue clearing methods. (**C**) Proton densities before and after application of each of the four tissue clearing methods. (**D**) Comparison of MFP changes between gray and white matter among the four tissue clearing methods. (**E**) Comparison of proton density changes between gray and white matter among the four tissue clearing methods. (**F**) Scatter plot of MFP versus proton density changes. Hydrogen index refers to normalized proton density with respect to the signal measured in the 1.5% agarose gel. Representative ROIs for gray matter and white matter are shown in Supplementary Fig. 4. The values used in the graphs in (**C**,**E**,**F**) represent means ± S.E.M.

For the comparative analysis of the changes in MFP in the cleared brain upon the use of the four different protocols, we created a color map for each cleared brain slice (Fig. 5A). In BABB clearing, the map of the MFP showed that color distribution was skewed towards dark blue. This means that the BABB technique provided the longest light transmission path and had the strongest overall clearing effect. In addition, tissue shrinkage was clearly observed after BABB clearing. In the case of Clear^T^ clearing, the map of the MFP displayed the widest color distribution and the lowest average, meaning that this technique was poor in clearing the lipid-rich brain regions. However, Clear^T^ preserved the original tissue structure relatively well. In both Scale and PACT clearing protocols, the map of the MFP showed moderate clearing effects. Moreover, tissue swelling was observed in both cases.

As mentioned above, the cortex and the corpus callosum are the two distinct brain regions in terms of their constituents: the cortex is a protein-rich domain (gray matter) whereas the corpus callosum is a lipid-rich domain (white matter). For this reason, we selected the entire cortex and the corpus callosum as shown in Supplementary Fig. 3 to quantify and compare the effects of the four clearing techniques as they were estimated by OCT and MRI. As shown in Fig. 5B,D, BABB clearing greatly increased the MFP both in the cortex and corpus callosum, showing that BABB has the strongest clearing effect. This is because BABB solution is organic, effectively dissolving lipids^3,4^. On the other hand, in Clear^T^, the lowest clearing effect in the corpus callosum was observed as compared to its effect in the cortex, as well as to the effect of the other three methods in the corpus callosum, because Clear^T^ solution is aqueous and does not significantly affect lipids^3,5^. In both Scale and PACT, the MFPs after clearing were similarly high in the corpus callosum, which shows their better clearing efficiency in lipid-rich regions. Compared to Clear^T^, Scale and PACT methods have a much stronger clearing effect in the lipid-rich corpus callosum because both protocols act by removing lipids^3,6^. In contrast, Scale and PACT have a weaker clearing effect than that of BABB. This outcome is consistent with previously reported results^3–6^, and thus we confirmed that our method can quantitatively examine the effect of existing clearing techniques.

We further estimated the proton density in the cerebral cortex and the corpus callosum in each brain slice to investigate the relationship between the regional differences of proton density and the optical clearing efficiency, upon the different clearing methods. As shown in Fig. 5C, the proton density was lower in the lipid-rich corpus callosum (0.874 ± 0.047; mean ± S.D.) than in the cortex (1.032 ± 0.037) in the brain slices before any clearing treatment. This regional difference in proton density became weaker after tissue clearing, and a more uniform proton density was observed across brain regions of different composition. In PACT and Scale clearing methods, the changes in the proton density were significantly higher in the corpus callosum as compared to the cortex (PACT: 0.323 ± 0.049, in the corpus callosum, 0.103 ± 0.057 in the cortex [p < 0.001]; Scale: 0.263 ± 0.052 in the corpus callosum, 0.128 ± 0.059 in the cortex [p < 0.001]) (Fig. 5E). This is in accordance with the larger amount of lipids contained in the corpus callosum, which was removed during clearing with Scale or PACT. In contrast, Clear^T^ and BABB involved dehydration which resulted in larger changes in the proton density in the cerebral cortex than in the corpus callosum (−0.190 ± 0.060 versus −0.100 ± 0.063 [p < 0.001], respectively for Clear^T^; −0.676 ± 0.148 versus −0.523 ± 0.142 [p < 0.001] respectively, for BABB) (Fig. 5E). In addition, the proton density difference between the cortex and corpus callosum was almost completely lost in BABB treated samples (cortex, 0.339 ± 0.135 versus corpus callosum, 0.349 ± 0.125 [p = 0.757]), but was maintained in Clear^T^ treated samples (cortex, 0.821 ± 0.033 versus −0.747 ± 0.038 [p < 0.001] (Fig. 5C). These results suggest that the extensive hydration and lipid removal upon BABB clearing leads to a more uniform chemical constitution in the brain samples, which is associated with the best clearing efficacy among the four methods.

The regional proton density changes were in excellent accordance with the optical clearing efficiency estimated by the measurement of the MFPs as shown in Fig. 5D,E. The lipid clearing methods, Scale and PACT, resulted in larger changes in proton density in the lipid-rich corpus callosum that correlated with larger increases in the optical MFPs. On the other hand, BABB and Clear^T^ showed larger dehydration in the cortex correlating with the observed higher increases in the optical MFPs. Therefore, we observed a good correlation between absolute (unsigned) change in proton density and the increase in the MFPs, estimated by OCT, in the white matter (corpus callosum) as presented in Fig. 5F.

## Discussion and Conclusions

Using OCT and MRI-based analyses, we compared the effect of different tissue clearing methods, in terms of proton density and MFP changes. Considering that these are two independent measurements derived using different imaging modalities, the observed correlations are informative in terms of understanding the way in which brain region-specific factors influence the clearing efficacy of the various existing protocols. Lipid clearing methods, such as Scale and PACT, were more effective in in lipid-rich white matter regions, with correspondingly higher levels of hyperhydration. On the other hand, Clear^T^ was more effective in the cortical gray matter region containing relatively more amount of water which was replaced with Clear^T^ solution. BABB showed the largest clearing effect amongst the four because it combines both extracting lipids and replacing water with a high refractive index solution. The changes in proton density observed in the cortical regions after the application of PACT and Scale were similar, correlating with the similar changes observed in the corresponding MFPs. The similar MFPs in the cortical regions after PACT, Scale, and Clear^T^ suggest that the refractive index matching of Clear^T^ is efficient for preventing light scattering by membrane lipids in the cortex. However, the aqueous Clear^T^ method showed worst clearing performance in the corpus callosum, i.e. the smallest changes in proton density and MFP. It indicates that the homogeneous scatterer distribution achieved by lipid-removal is critical for the optical clearing of lipid-rich white matter regions.

There are a few methodological issues to be noted. First, the proton density MRI in the present study quantifies the amount of water in the sample primarily, and does not capture change in lipid component directly. Instead, the proton density MRI reflects hyperhydration associated with lipid removal in Scale and PACT clearing. The lengthened T_2_ values in gray and white matter regions, as shown in Supplementary Fig. 4, confirmed the contribution of lipid washout as well, in consistence with previous results on PACT^22^. Accordingly, both of the larger changes in proton density and T_2_ values in white matter regions upon Scale and PACT clearings also corroborate the fact that the hyperhydration-associated change was more profound in lipid-rich regions. Second, Scale and PACT cleared samples were embedded in 1.5% agarose gel for MRI scan in the present study because the cleared brain sample was very soft and fragile due to hyperhydration. The 1.5% agarose gel also served as a baseline measurement for normalizing proton density MRI signal intensity. Scale and PACT solutions are aqueous but there may be potential exchange of water and salt between the cleared brain sample and the surrounding agarose gel (despite no visible sign of deformation or impaired clearing after gel-embedding in the present study). In addition, we omitted a final step of PACT protocol, immersing the cleared sample in a refractive index matching solution, because we focused to compare lipid removal effect of PACT directly in both OCT and MRI.

For the direct comparison of proton density changes between different clearing methods in the cortical region, the effect of the clearing solution needs to be considered for Clear^T^ and BABB results. For example, the reduction in proton density in the brain tissue after Clear^T^ application is the result of the replacement of extracellular water with formamide, which has a relatively low proton density as shown in the colored proton density map, surrounding the brain slice (inside the syringe; Fig. 4). Similar proton densities and T_2_ values between the cortical brain tissue and the surrounding formamide solution indicate the thorough absorption of the clearing solution in the gray matter. The larger proton density change (reduction) of Clear^T^ data point, even with similar optical MFP values with respect to Scale/PACT shows the influence of the clearing solution, as shown in the scatter plot for the gray matter in Fig. 5F. Considering that the T_2_ shortening is similar between gray and white matter after Clear^T^, as shown in Supplementary Fig. 4, it is apparent that the formamide solution is also perfused in the white matter. On the other hand, the smallest proton density change in the white matter, after Clear^T^, ascertains that lipids remained intact. Thus, the absolute proton density change in the white matter is proportional to the level of lipid removal, which is accordingly correlated with the optical MFP changes across the different clearing methods, evaluated in this study.

Technically, the OCT-based MFP measurement utilizes non-invasive optical tomographic imaging with scattering contrast, thus the clearing process and the enhancement of imaging depth can be thoroughly visualized. In particular, mapping of the optical properties on the en-face image of the brain slice offers quantitative and comprehensive information simultaneously. OCT-based investigation of cleared tissues can also provide information on the unexpected structural deformation of outer and inner parts of the tissue. Thus, the platform of the OCT-based analysis is a promising tool for optimizing and monitoring the clearing protocol not only for brain slices, but also for other cleared tissues such as the kidney and the heart. In a case that slicing of the sample for OCT quantification of MFP is not applicable, the MRI proton density measurement can also be utilized to estimate the clearing efficacy in lipid-rich tissues in conjunction with the proportional relationship with the optical MFP change observed in the present study.

In conclusion, our OCT measurement of the MFP could quantitatively assess regional variations in the clearing effect within the brain tissue. Each clearing method showed different clearing efficiency for protein-rich gray matter region and lipid-rich white matter region in the brain depending on its mechanism of action. The optical transparency measurement of OCT was in good agreement with the regional proton density changes calculated by independent MRI measurements. Our results demonstrated the feasibility of quantitative mapping of optical clearing efficacy and accompanying proton density change in conventional tissue clearing techniques using OCT and MRI. It is worthwhile to note that next generation alternatives, which are known to be more efficient (SCALE/s^25^, iDISCOplus^26^, uDISCO^27^, Adipoclear^28^) are being developed, and OCT/MRI imaging based quantification can be extended to updated tissue clearing methods. In addition, other water/fat MRI techniques such as proton density fat fraction (PDFF) and magnetic resonance spectroscopy (MRS) can be also utilized for more elaborate measurement of water and lipid contents in future studies^29,30^. As these imaging techniques can be used for the quantitative comparison of various tissue clearing methods for tissues of varied composition, they may provide essential quantitative metrics for the development and evaluation of optimized clearing methods for different biological samples.

## Supplementary information

Supplementary Information
