# Supplementary material for: Quantitative assessment of regional variation in tissue clearing efficiency using optical coherence tomography (OCT) and magnetic resonance imaging (MRI): A feasibility study
Source: Sci Rep. 2019 Feb 27;9:2923. doi: 10.1038/s41598-019-39634-z (PMC6393517; doi:10.1038/s41598-019-39634-z)
Supplement: Supplementary file 1 — Supplementary Information [file 41598_2019_39634_MOESM1_ESM.docx]

Quantitative assessment of regional variation in tissue clearing efficiency using optical coherence tomography (OCT) and magnetic resonance imaging (MRI): A feasibility study

Kwangyeol Baek^1,†^, Sunwoo Jung^1,†^, Junwon Lee^1^, Eunjung Min^2^, Woonggyu Jung^1,*^, Hyungjoon Cho^1,*^

^1^Department of Biomedical Engineering, Ulsan National Institute of Science and Technology (UNIST), Ulsan, South Korea

^2^The Rowland Institute at Harvard, 100 Edwin H. Land Blvd. Cambridge, MA 02142, USA
*Corresponding authors: [wgjung@unist.ac.kr](mailto:wgjung@unist.ac.kr), hjcho@unist.ac.kr

^†^these authors contributed equally to this work

**SUPPLEMENTARY FIGURES AND FIGURE LEGENDS**

**
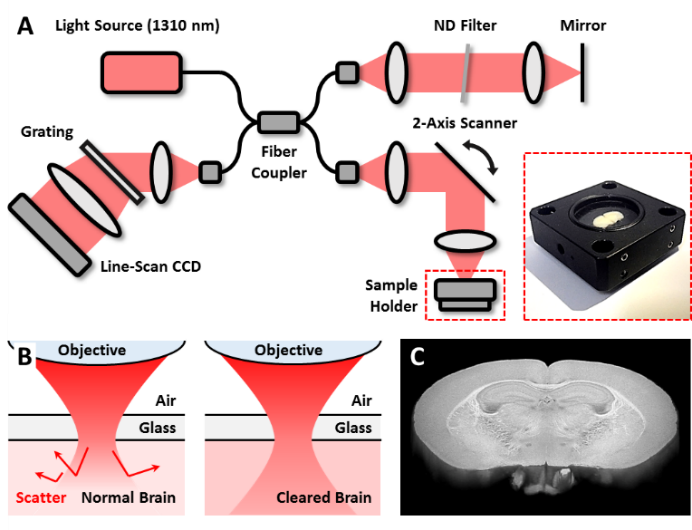
**

**Supplementary Fig. 1.** Schematic overview of the OCT-based method for the monitoring of tissue optical properties. (A) Schematic diagram of a fiber-optic spectral-domain OCT (SD-OCT) system with a specially designed sample holder (photograph in the inset on the right). (B) Cross-sectional illustration showing the effect of tissue clearing on turbid biological tissue in the sample holder. In the cleared brain, light scattering is reduced and thus light transmission is improved. (C) Representative 3D OCT image of a coronal brain slice. ND filter, neutral density filter; CCD, charge-coupled device.


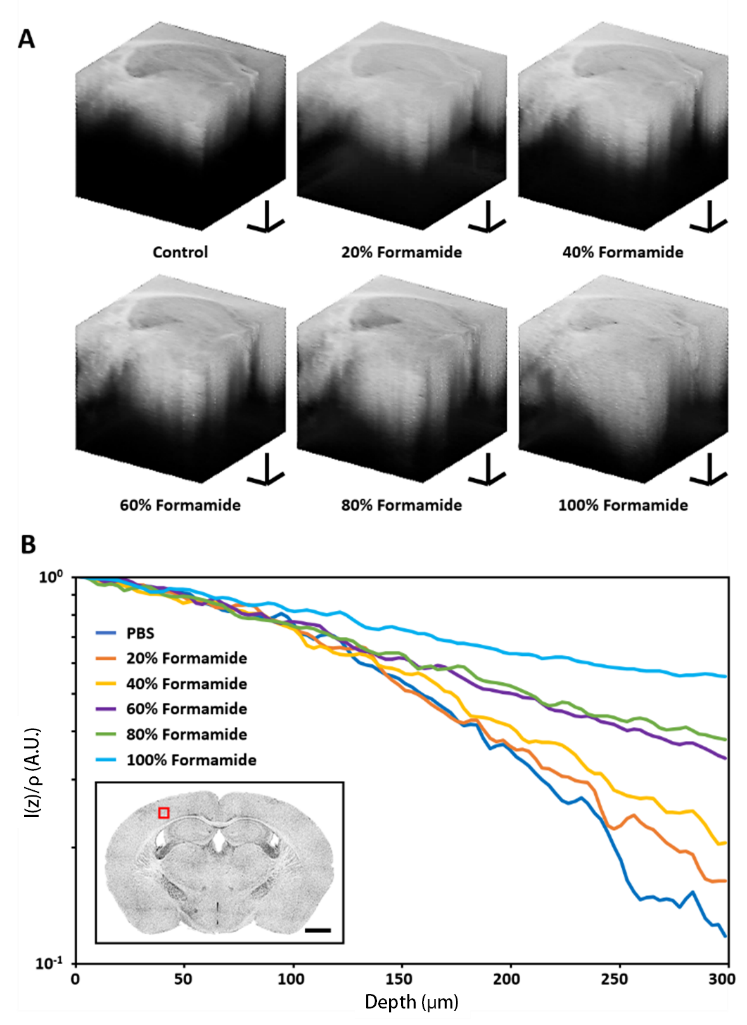


**Supplementary Fig. 2.** Enhancement of OCT imaging depth in the cleared brain slice. (A) A series of 3D OCT images after treatment with solutions of increasing formamide concentrations showing that clearing reduces light scattering. The volume of these images is 2.5 × 2.5 × 1.7 mm^3^. Scale bars represent 500 μm. (B) OCT signal variations along tissue depth in relation to the degree of tissue clearing. Inset shows an OCT reflectivity image. Plotted data are averaged OCT signals in the cortex ROI delineated by the red rectangle. Scale bar represents 2 mm. A.U., arbitrary units; I(z), light intensity at distance z; ρ, reflectivity.

**
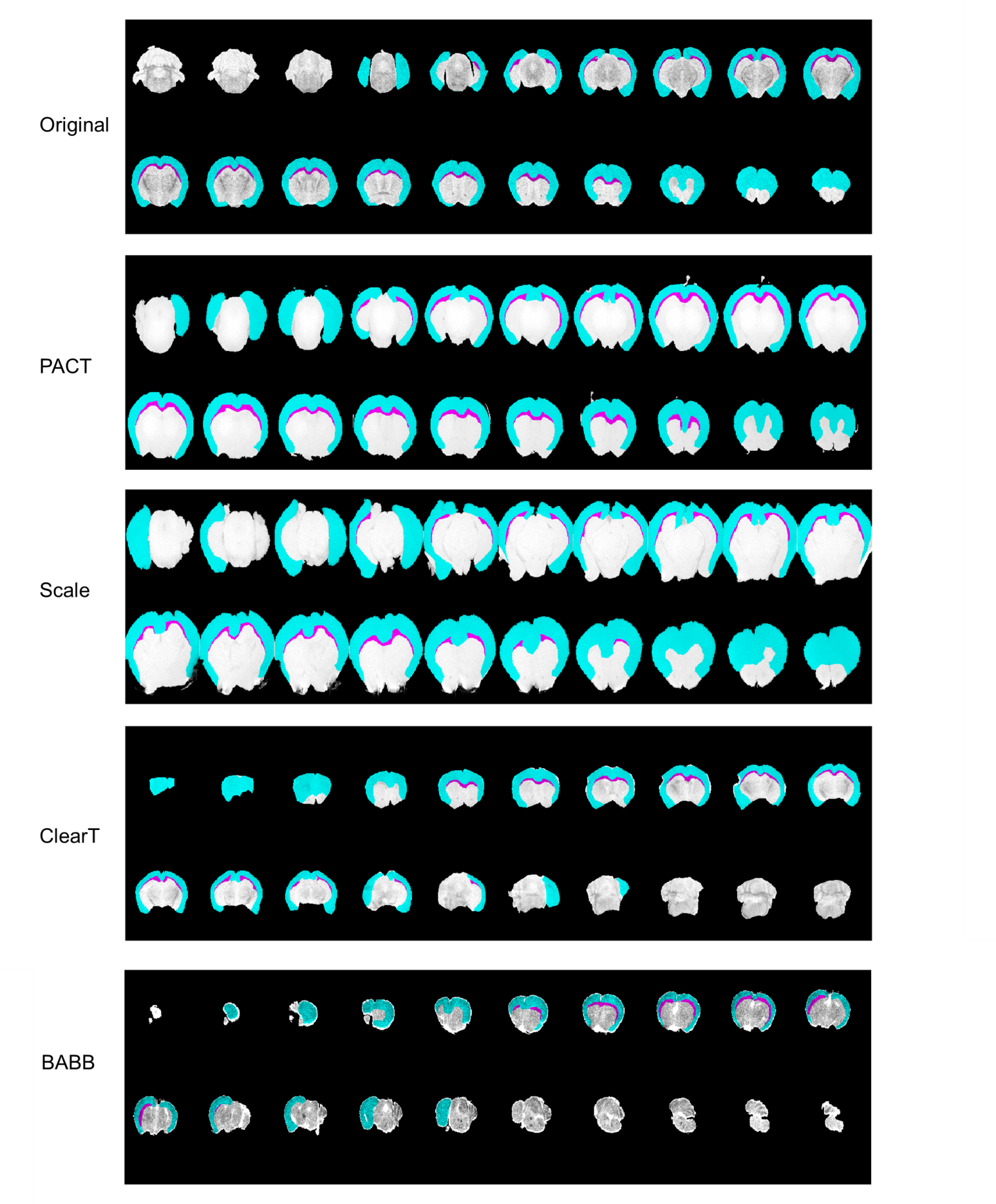
**

**Supplementary Fig. 3.** Examples of cortex (gray matter) and corpus callosum (white matter) ROIs for each of the four clearing methods used in the MRI data analysis (Representative samples). Correspondingly, entire cortex and corpus callosum ROIs are used for separate OCT data analysis shown in Figure 5.


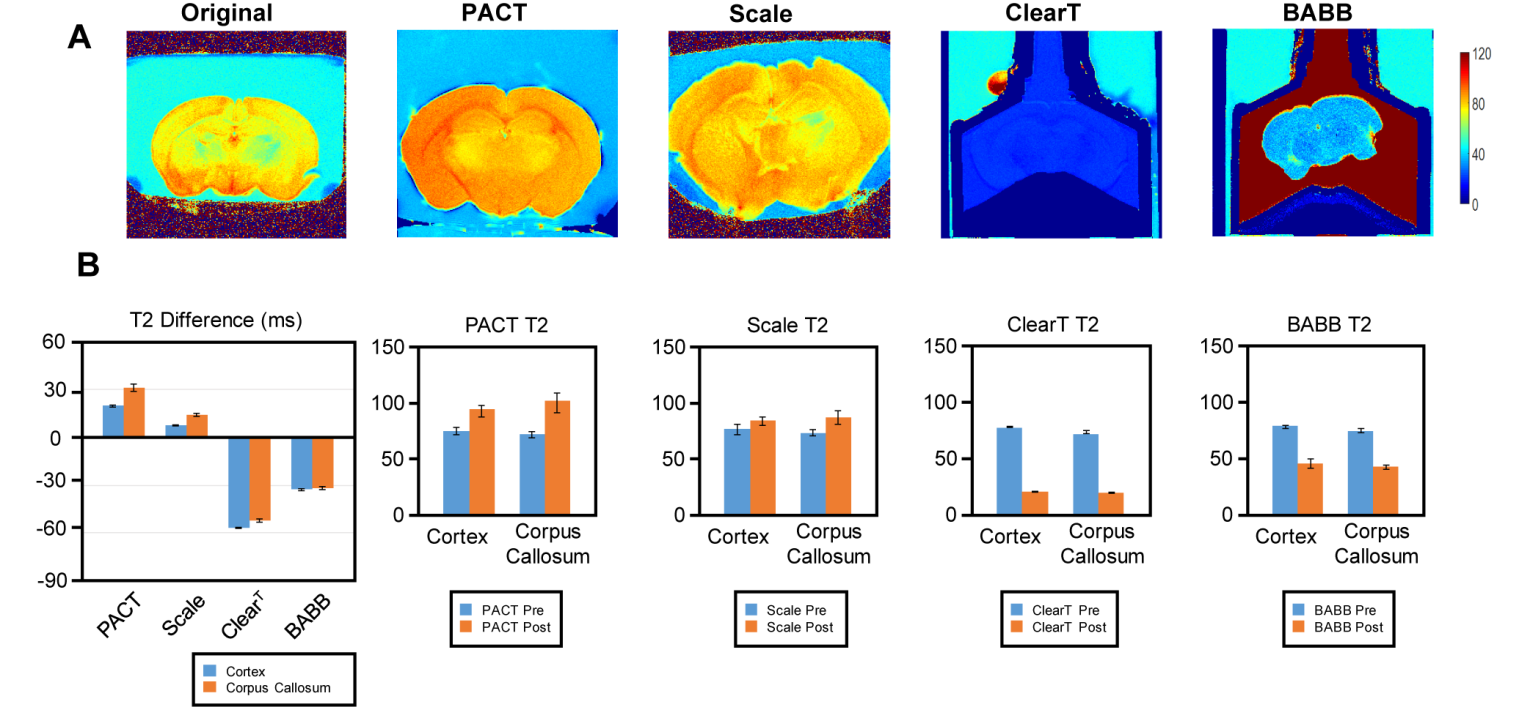


**Supplementary Fig. 4.** MRI T_2_ relaxation time measurements in brain samples before and after tissue clearing with each of the four different protocols. (A) T_2_ map of original (before clearing) and cleared brains; (B) T_2_ changes upon the different clearing methods in the cortex and the corpus callosum. The values used in the graphs in (B) represent means ± SD.


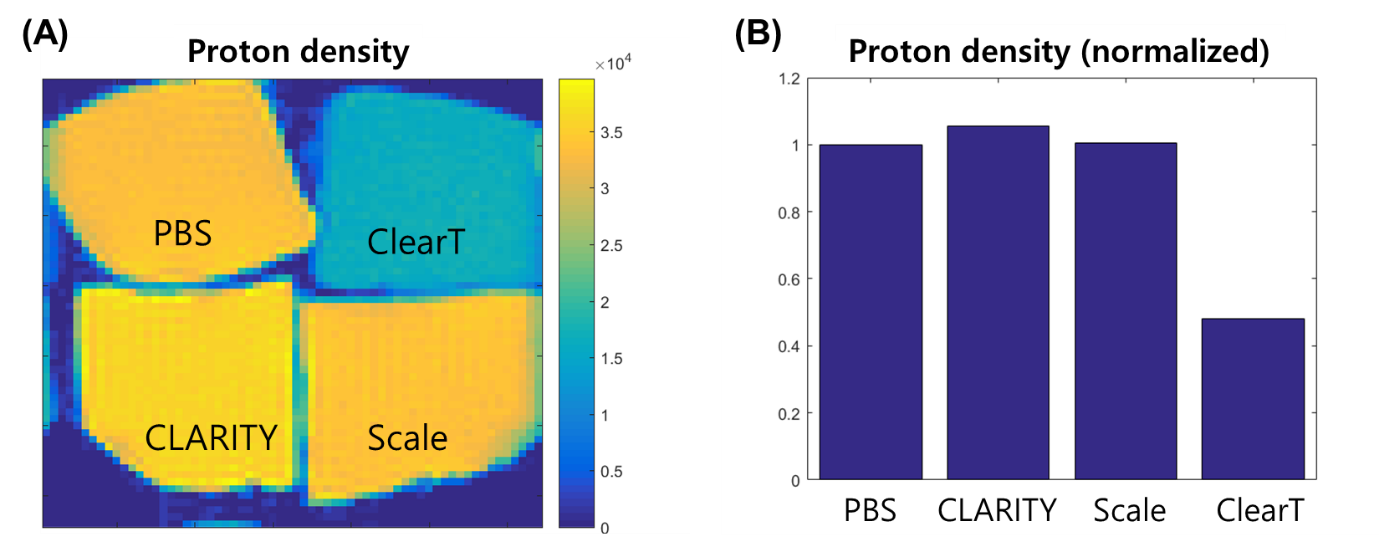


**Supplementary Fig. 5.** (A) MRI proton density maps of agarose gel samples equilibrated with respective clearing solutions and water. (B) Normalized proton density values were shown correspondingly. Proton density was substantially lower in agarose gel which was immersed in ClearT solution. Proton density in agarose gel with CLARITY and Scale solution was increased by 6.6% and 1.0% compared to the baseline (agarose gel immersed in PBS solution). Thus, we decided not to equilibrate the agarose gel-embedded samples (CLARITY and Scale) with clearing solutions in the MRI experiment because agarose gel served as a reference for normalizing MR proton density measurements.
